# An exceptionally rare case of a giant parathyroid adenoma with carcinoma-like presentation

**DOI:** 10.1007/s42000-025-00627-5

**Published:** 2025-01-20

**Authors:** Paraskevi Kazakou, Dionysios Vrachnis, Stavroula A. Paschou, Konstantinos Nastos, Helen Sarlani, Kanella Kantreva, Katerina Stefanaki, Theodora Psaltopoulou, George Kyriakopoulos, Penelope Korkolopoulou, Katerina Saltiki

**Affiliations:** 1 Endocrine Unit and Diabetes Centre, Department of Clinical Therapeutics, Alexandra Hospital, School of Medicine, National and Kapodistrian University of Athens, Athens, Greece; 2 Third Department of Surgery, School of Medicine, “Attikon” University General Hospital, National and Kapodistrian University of Athens, Athens, Greece; 3 Department of Pathology, Medical School, National and Kapodistrian University of Athens, 75 Mikras Asias Str., Athens, 11527 Greece; 4 Department of Pathology, Evangelismos Hospital, Athens, Greece

**Keywords:** Giant parathyroid adenoma, Hypercalcemia, Hyperparathyroidism, Hungry bone syndrome, Parathyroid carcinoma

## Abstract

Giant parathyroid adenoma (GPA) is an extremely rare cause of primary hyperparathyroidism (PHPT) and may sometimes mimic parathyroid carcinoma (PC). Parathyroid carcinoma is also a very rare entity. Both preoperative and postoperative diagnosis of the two conditions remains a challenge. The purpose of this article is to present the diagnostic and therapeutic approach used for a 76-year-old female patient with a GPA measuring 5.4 × 2.3 cm, mimicking PC. The patient was referred to our clinic for the management of severe hypercalcemia revealed during the neurological evaluation of psychiatric and cognitive symptoms, confusion, weakness, and bone pain. PHPT was confirmed based on the patient’s biochemical profile, which showed extremely high levels of serum calcium and parathyroid hormone (PTH). Wholebody computed tomography revealed a large nodule below the inferior pole of the right lobe of the thyroid gland and no further pathology in other organs. En bloc resection of the tumor with removal of the ipsilateral hemithyroid and other involved tissues was performed. Histopathological evaluation was diagnostic for a GPA. Post-surgery hungry bone syndrome (HBS) developed and was treated. However, the patient succumbed 3 weeks later due to septic shock. GPA is an exceptionally rare endocrine tumor that should be suspected along with PC in patients with significantly elevated levels of PTH and calcium, and/or palpable neck mass. In our case, diagnosis was based principally on histopathological examination together with clinical presentation, biochemical profile, and imaging studies. Resection of the tumor remains the treatment of choice.

## Introduction

Primary hyperparathyroidism (PHPT) is a common endocrine disease with an incidence of approximately 25/100,000 people per year, increasing with age and presenting a female preponderance of 3:1, peaking in the seventh decade of life. The solitary parathyroid adenoma is the principal cause of PHPT in 80 to 85% of cases. Multiple adenomas, parathyroid hyperplasia, and parathyroid carcinoma (PC) account for 10 to 15%, 5%, and 1% of cases, respectively [1, 2]. PHPT has characteristic clinical signs and symptoms such as osteoporosis and pathological fractures, gastrointestinal disturbances, neuropsychiatric disorders, neuromuscular manifestations, and nephrolithiasis or nephrocalcinosis, eventually complicated with urinary infections and kidney failure, which can be attributed to elevated levels of serum calcium and parathyroid hormone (PTH).

The typical parathyroid gland weighs 50 to 70 mg and, usually, parathyroid adenomas are small in size and have a median weight of 600 mg. Adenomas weighing more than 3.5 g are extremely rare and are classified as giant parathyroid adenomas (GPAs). GPAs and PCs, both uncommon causes of PHPT, may share several common characteristics without definitive diagnostic predictors, which makes it challenging to differentiate them, especially in cases of non-invasive metastatic disease [3–5]. It is crucial to distinguish PC from benign adenomas or hyperplasia since initial radical surgery offers the best chance for cure [6].

Herein, we report a rare case of a GPA with extremely high levels of calcium and PTH and describe the diagnostic and therapeutic difficulties that arose by illustrating the differential aspects between the GPA and PC.

## Case description

A 76-year-old female was referred to our department from the nearby university psychiatric hospital due to severe hypercalcemia. She had been admitted for severe psychiatric disorders, rapidly deteriorating during the past 2 months (namely, confusion, depression, disorientation, dementia, and organic psychosis). Her past medical history revealed a meningioma (at 20 years old), Hashimoto’s thyroiditis with hypothyroidism under levothyroxine treatment, hypertension under losartan treatment, mild chronic kidney disease (CKD), and nephrolithiasis diagnosed 2 years previously and osteoporosis during the last 6 years under treatment with denosumab and calcium until 6 months before admission. No history of neck irradiation or familial endocrine disease was reported. She also complained of fatigue, severe asthenia, and bone pain along with confusion and mental disorientation for a period of 20 days before admission. Of note, she had started treatment with memantine hydrochloride for Alzheimer’s disease 1 month before admission.

On physical examination, her blood pressure was 120/75 mm Hg, pulse rate 94 beats/min, respiratory rate 25/min, temperature 36.7 ^o^C, and oxygen saturation 86–92%. She was confused. There was a palpable firm painless lump in the right thyroid lobe without palpable cervical adenopathy. Her pulse rate was regular, lungs were clear to auscultation, and abdomen was non-tender. The rest of the examination findings were normal.

Laboratory test results revealed severe hypercalcemia (Ca = 24.3 mg/dl, reference range (rr): 8.5–10.2), hyperphosphatemia (*P* = 5.1 mg/dl, rr: 2.5–4.5), mild hypomagnesemia (Mg = 1.5 mg/dl, rr: 1.6–2.3), elevated serum creatinine (Cr = 1.53 mg/dl), elevated C-reactive protein (CRP = 31 µg/ml, rr: 0–5), increased white blood count (WBC = 22.700/µL), and low TSH level (TSH = 0.271 µU/ml, rr: 0.4-4). Serum alkaline phosphatase (ALP) remained within normal limits. The serum albumin level was also within normal limits (Alb = 4.4 g/dl, rr: 3.5-5) and the estimated glomerular filtration rate (eGFR) was 33 ml/min/1.73m^2^.

### Investigation and treatment

Subsequently, serum PTH levels were measured for the differential diagnosis between hypercalcemia of malignancy and severe primary hyperparathyroidism. Serum PTH was remarkably elevated (PTH = 2657 pg/ml, rr: 15–65), 41 times above the upper normal limit. Thus, the extremely high levels of both calcium and PTH led to the diagnosis of a very severe case of PHPT. The differential diagnosis included parathyroid adenoma, parathyroid hyperplasia, and, mainly, PC.

**Fig. 1:**
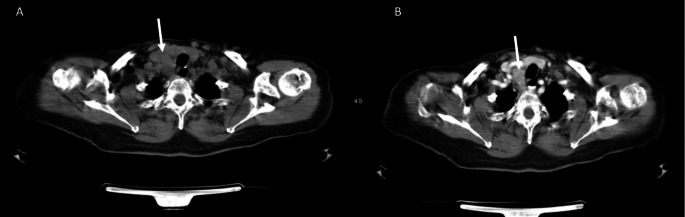
CT scan of the neck. The arrows show the presence of a parathyroid gland nodule of 2 × 3.3 cm in the right paratracheal region with extension to the superior mediastinum. (**A**) before and (**B**) after contrast agent

Since the patient had a hypercalcemic crisis, aggressive hydration with isotonic fluids accompanied by intravenous furosemide and prednisolone, and subcutaneous denosumab, were administered. Furthermore, a whole-body computed tomography (CT) scan was performed which revealed a mass below the right inferior pole of the thyroid gland measuring 2 × 3.3 cm with an extension in the upper mediastinum and nephrolithiasis in the left kidney (Fig. 1). CT scan also showed nephrolithiasis. Meanwhile, broad-spectrum antibiotic treatment with piperacillin-tazobactam was started intravenously because of leukocytosis and elevated CRP.

The second day after admission, the patient’s renal function rapidly worsened and she required dialysis. Treatment with cinacalcet (90 mg x4) was initiated and the patient underwent a second dialysis session. However, she was rendered hemodynamically unstable and lethargic, while her oxygen saturation decreased. Norepinephrine IV was administered and she was subsequently intubated and admitted to the intensive care unit (ICU). As there was a strong clinical suspicion of PC in view of the lesion size, as well as the excessively high calcium and PTH levels, an emergency surgery was planned.

**Fig. 2:**
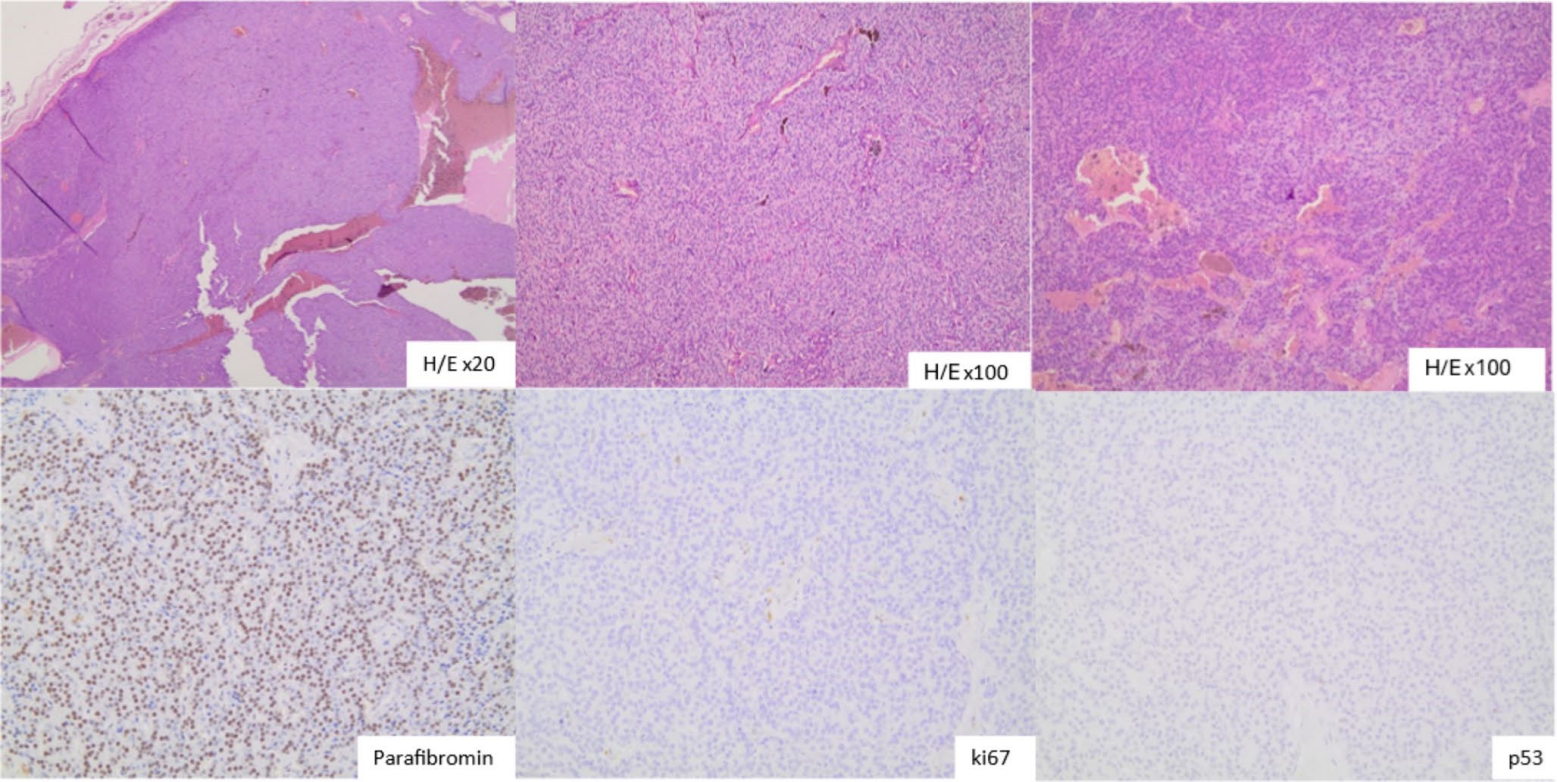
Section showing parathyroid adenoma composed of uniform chief cells without prominent mitotic activity or nuclear atypia. There is no capsular invasion or extension into adjacent fat. Stroma is sparse. There are areas of cystic change. Parafibromin expression: Preserved, ki67: (-), p53: (-)

The tumor was resected en bloc with the surrounding tissue, including the right thyroid lobe and the right recurrent laryngeal nerve to which it was firmly attached. Histological examination of the surgical specimen confirmed the diagnosis of a GPA with no signs of malignancy, measuring 5.4 × 2.3 cm and weighing 11 g. Microscopy showed a proliferation of chief cells without atypia or fibrous bands, with focal areas of cystic and hemorrhagic degeneration. There was no capsular invasion or extension into adjacent fat. Immunohistochemical staining for parafibromin was diffusely positive. Immunohistochemical staining of ki-67 and p53was negative (Fig. 2).

### Outcome and follow-up

Within the first 3 postoperative days, serum Ca and P concentrations gradually normalized. Serum PTH also returned to normal levels (PTH = 31 pg/ml) (Table 1). However, the antibiotic therapy was changed to meropenem along with vancomycin due to worsening infection (CRP = 369 µg/ml, rr: <5).

**Table 1:** Laboratory evaluation

	At presentation	Pre-surgery	Post-surgery Day 4	Post-surgery Day 6 under calcium iv	Ref. range
White blood cells	22.700	22.000	23.400	17,700	5000-10.000 cells/microL
CRP	31	53	370	317	< 5 µg/ml
Calcium	24.3	18.2	6.4	6.5	0.8.5–10.2 mg/dl
Albumin	4.4	2.2	1.7	2.1	3.5–5 g/dl
Phosphate	5.1	4	3	2.8	2.5–4.5 mg/dl
Parathyroid hormone	2657	-	31	30	15–65 pg/ml
Creatinine	1.53	1.18	2.77	1.81	< 1.30 mg/dl

On day 4 postoperatively, hungry bone syndrome (HBS) was diagnosed with a low serum calcium level (Ca = 6.4 mg/dL), while the serum albumin level was 1.7 g/dL. Corrected calcium level was 8.2 mg/dL. The syndrome was initially treated with intravenous calcium (100 mL of 10% calcium gluconate diluted in 1000 mL 5% dextrose) along with cholecalciferol and alfacalcidol in order to maintain serum calcium levels within the lower normal range. Οn day 6 postoperatively, corrected calcium was maintained at 8 mg/dl under intravenous calcium treatment (Table 1). Unfortunately, the patient passed away 3 weeks after surgery due to septic shock and multiorgan failure.

## Discussion

We present a rare case of a rapidly progressive GPA in an elderly woman who was initially admitted with extremely high levels of serum calcium and PTH as well as a rapidly deteriorating level of consciousness accompanied by the development of severe psychiatric disorders.

GPAs and PCs are both very rare entities. Their preoperative and pathological diagnosis is challenging, particularly in the absence of invasion of adjacent structures and distant metastases [3, 5, 7, 8]. The signs and symptoms of severe hypercalcemia are common to both PC and GPA. Clinical features due to infiltration of vital organs by tumor mass are rare in PC [9]; in most cases it is an indolent, slow growing but tenacious malignant tumor [6]. It is reported that the classical target organs of PTH, the kidney and skeleton, are affected with greater frequency and severity in PC. Bone pain and pathological fractures occur in 34–73% of cases, while renal pathology, such as polyuria, nephrolithiasis, and renal colic, is frequent in 32–70% [7, 10]. Of note, in our case, the main symptoms, apart from bone pain, nephrolithiasis, and kidney failure, were psychiatric disorders (depression, dementia, and organic psychosis), which developed in quite a short period. Concerning demographics, the age and the female: male ratio are higher in PA than in PC. However, there is a considerable overlap. It is reported that the average age of occurrence of PC is in the fifth decade, while our patient was in her 70s [5, 9].

Most patients with PC present with severe hypercalcemia (65–75%), generally above 14 mg/dl or 3–4 mg/dl above the upper limit of normal, while PTH levels are usually 5 to 10 times higher than normal. In a review by Obara et al., in two-thirds of 133 patients, mean serum calcium was 15.0 mg/dl (range 10.0–24 mg/dl) and serum PTH levels > 5 times the upper limit of the normal range [6]. Concerning PAs, most authors have reported a positive correlation between adenoma weight and the biochemical abnormalities. Indeed, a cohort of 378 PHPT cases showed a significant positive correlation of both preoperative calcium and PTH values with adenoma dimensions and weight [11]. Similarly, a retrospective study by Leong et al. confirmed these findings and suggested that PTH might be a better predictor of PA weight than serum calcium levels in patients with PA [12]. By contrast, in a recent systematic review of 65 patients with GPAs with hypercalcemia (mean 14.06 mg/dl, range: 10.4–23) and increased PTH levels (> 14 times above the upper limit of the normal reference), no correlation between the GPA size or weight and PTH levels was found [13]. In any case, in general, the symptoms of hypercalcemia are more frequent and severe in PC than in PA [5]. Of note, a study by Spanheimer et al. showed that GPAs are more frequently asymptomatic than non-giant PA despite higher levels of calcium and PTH [14].

Concerning our patient, the initial serum calcium concentration was notably elevated at 24.3 mg/dl along with a PTH level 41 times above the upper normal limit with symptoms indicating hypercalcemic crisis, which is not usual even in giant adenomas. A hypercalcemic crisis is a life-threatening endocrine emergency characterized by extremely high serum calcium concentration (> 14 mg/dl) with concomitant multiorgan dysfunction (gastrointestinal and neurological symptoms, acute renal failure, and cardiac rhythm abnormalities) [15]. Our patient exhibited renal and neurological dysfunctions, expressed as markedly elevated creatinine levels and impaired consciousness. Thus initially, in the present case, the clinical and biochemical profile raised a strong suspicion of PC.

To our knowledge, this is the first case reported in the literature of GPA with such high levels of serum calcium and PTH. Another exceptional case of a GPA mimicking PC and measuring 6.5 cm with milder preoperative hypercalcemia of 12.5 mg/dl and PTH of 2.747 pg/ml was published in 2020 [16]. The differential diagnosis of these entities is based on the clinical features and the laboratory findings. Preoperative fine-needle aspiration (FNA) is not recommended because cytology cannot distinguish between benign and malignant disease. In the case of PC, there have also been reports of tumor seeding along the biopsy tract [17, 18]. Imaging modalities such as neck ultrasound, CT scan, magnetic resonance imaging (MRI), and ^99^mTc-MIBI (sesta-MIBI) scintigraphy are mandatory in order to locate the tumor and metastatic lesions. In our patient, a neck CT scan demonstrated a large heterogenous solid lesion of 2 × 3.3 cm below the right inferior pole of thethyroid gland extending into the upper mediastinum.

Differentiating between an adenoma and PC is difficult as no specific imaging characteristics exist [19]. Features such as irregular margins, pathological lymph nodes, and invasion of the adjacent structures may be indicative of PC [7, 9].

Surgery remains the gold standard procedure for the treatment as well as for the final differential diagnosis of PC vs. GPA. Therefore, our patient underwent en bloc right lobe thyroidectomy, parathyroidectomy, and removal of the right recurrent laryngeal nerve due to apparently tumor infiltration. In the case of PC, en bloc dissection of the tumor together with the lateral thyroid lobe, ipsilateral parathyroid, and other adjacent tissues invaded by the tumor (such as paratracheal alveolar and lymphatic tissue, the thymus or some of the neck muscles, and the recurrent laryngeal nerve) is associated with the best prognosis [6, 20].

Histopathological distinction between benign and malignant parathyroid tumors is also difficult. Macroscopically PC presents as a firm, lobulated mass surrounded by dense fibrous tissue that invades or adheres to surrounding structures including the thyroid gland, the strap muscles, the recurrent laryngeal nerve, esophagus, and trachea [6]. GPAs tend to be bright reddish or brown, soft, and ovoid. However, up to 20% of GPAs and PCs can be misclassified if only these parameters are taken into account [21]. Microscopically, capsule, blood vessel and lymph invasion, stromal calcifications, fibrous trabeculae, enlarged nuclei, and strong mitotic activity are considered to be signs of malignancy according to the histopathological criteria of Shantz and Castleman [22]. Furthermore, the loss of staining for parafibromin, a protein coded by the HRPT2 gene (involved in the pathogenesis of PC), and increased values of Ki-67% are two additional elements indicating a PC in the majority of cases [5, 16]. Fortunately, in the present case, to our surprise, despite the hypercalcemic crisis with markedly elevated levels of calcium and PTH and the exceptionally firm tumor found during surgical excision, the histological examination established the diagnosis of a GPA measuring 5.4 cm x2.3 and weighting 11 g, with no atypias, positive parafibromin staining or negative Ki67 and p53 staining. In addition, there was partial cystic and hemorrhagic degeneration. Cystic transformation is an unusual presentation. Cystic parathyroid lesions are very uncommon, accounting for less than 0.01% of all neck masses [8, 23]. In our patient, focal areas of cystic degeneration of the gland were noted, probably due to the huge dimensions and longtime evolution of the disease. On the other hand, the unusually rapid onset of hypercalcemia with severe symptoms in this case could be attributed to a possible acute hemorrhage of the lesion.

Post-surgery restoration of normocalcemia is a clear sign that the entirety of hyperfunctioning tissue has been excised. During the early postoperative period, however, there should be strict monitoring of serum calcium level due to the risk of symptomatic hypocalcemia arising from HBS, as in our patient. Hypocalcemia due to HBS suggests that the surgery has been successful [9]. It is observed in 13–30% of parathyroid surgery cases [24] and some studies suggest the use of bisphosphonates prior to surgery in order to prevent this post-operative outcome [25]. The hypocalcemia of HBS may be rapid, severe (serum calcium concentration < 8.4 mg/dl), and prolonged and can be associated with hypophosphatemia and hypomagnesemia. It is exacerbated by low PTH levels following parathyroidectomy in patients with severe PHPT and preoperative high bone turnover. HBS treatment requires high doses of calcium (6 and 12 g/day) as well as active metabolites of vitamin D and replenishment of magnesium stores as needed. Intravenous calcium replacement may be temporarily necessary in severe forms of HBS, as observed in our case [24, 26].

## Conclusion

This report presents an exceptionally rare, rapidly progressing case of GPA mimicking a PC in terms of glandular size and profound hypercalcemia and PHPT. The most effective therapy is complete resection of the lesion. For this reason, both preoperative suspicion and intraoperative recognition are of paramount importance. Postoperatively, HBS is potentially a serious condition to which clinicians should be highly attuned. The need for increased clinical vigilance should be underlined. In our case, despite the successful surgical excision of the GPA and the effective management of the HBS, the patient succumbed 3 weeks after surgery because of septic shock.
